# Human Discrimination and Categorization of Emotions in Voices: A Functional Near-Infrared Spectroscopy (fNIRS) Study

**DOI:** 10.3389/fnins.2020.00570

**Published:** 2020-06-05

**Authors:** Thibaud Gruber, Coralie Debracque, Leonardo Ceravolo, Kinga Igloi, Blanca Marin Bosch, Sascha Frühholz, Didier Grandjean

**Affiliations:** ^1^Neuroscience of Emotion and Affective Dynamics Lab, Department of Psychology and Educational Sciences and Swiss Center for Affective Sciences, University of Geneva, Geneva, Switzerland; ^2^Cognitive Science Center, University of Neuchâtel, Neuchâtel, Switzerland; ^3^Department of Neuroscience, Faculty of Medicine, University of Geneva, Geneva, Switzerland; ^4^Geneva Neuroscience Center, University of Geneva, Geneva, Switzerland; ^5^Department of Psychology, University of Zurich, Zurich, Switzerland; ^6^Neuroscience Center Zurich, University of Zurich and ETH Zürich, Zurich, Switzerland; ^7^Center for Integrative Human Physiology, University of Zurich, Zurich, Switzerland

**Keywords:** categorization, discrimination, emotion, fNIRS, prosody

## Abstract

Functional Near-Infrared spectroscopy (fNIRS) is a neuroimaging tool that has been recently used in a variety of cognitive paradigms. Yet, it remains unclear whether fNIRS is suitable to study complex cognitive processes such as categorization or discrimination. Previously, functional imaging has suggested a role of both inferior frontal cortices in attentive decoding and cognitive evaluation of emotional cues in human vocalizations. Here, we extended paradigms used in functional magnetic resonance imaging (fMRI) to investigate the suitability of fNIRS to study frontal lateralization of human emotion vocalization processing during explicit and implicit categorization and discrimination using mini-blocks and event-related stimuli. Participants heard speech-like but semantically meaningless pseudowords spoken in various tones and evaluated them based on their emotional or linguistic content. Behaviorally, participants were faster to discriminate than to categorize; and processed the linguistic faster than the emotional content of stimuli. Interactions between condition (emotion/word), task (discrimination/categorization) and emotion content (anger, fear, neutral) influenced accuracy and reaction time. At the brain level, we found a modulation of the Oxy-Hb changes in IFG depending on condition, task, emotion and hemisphere (right or left), highlighting the involvement of the right hemisphere to process fear stimuli, and of both hemispheres to treat anger stimuli. Our results show that fNIRS is suitable to study vocal emotion evaluation, fostering its application to complex cognitive paradigms.

## Introduction

While the majority of the studies investigating cognitive processes in cortical regions have relied on functional magnetic resonance imaging (fMRI) or electroencephalography (EEG), the use of functional near-infrared spectroscopy (fNIRS) as an imaging technique has developed over the last 25 years (Chance et al., 1993; Hoshi and Tamura, 1993; Kato et al., 1993; Villringer et al., 1993; Boas et al., 2014; Buss et al., 2014; Homae, 2014). Similar to fMRI, fNIRS is a non-invasive and non-ionizing method that investigates the brain hemodynamics (Boas et al., 2014). Using the principle of tissue transillumination, fNIRS indirectly measures via near-infrared light the oxygenated hemoglobin (Oxy-Hb) and deoxygenated hemoglobin (Deoxy-Hb) sustaining the hemodynamic response function (HRF). In effect, optical property changes assessed by two or more wavelengths between the optical fibers detecting and receiving the near-infrared light provide an indirect measure of cerebral Oxy-Hb and Deoxy-Hb; an increase of Oxy-Hb concentration suggests that the area considered is more active during a particular paradigm compared to a control condition (Mandrick et al., 2013; Scholkmann et al., 2014). Research findings using fNIRS suggest that this method can be an appropriate substitute to fMRI to study brain processes related to cognitive tasks (Cui et al., 2011; Scholkmann et al., 2014) with a more realistic approach (Strait and Scheutz, 2014). Despite a lower spatial resolution than fMRI, fNIRS has indeed a high temporal resolution, and is particularly interesting because of its low-cost and high portability, allowing for instance one to measure participants while they are engaged in a sport activity (Piper et al., 2014). The fNIRS signal is also less sensitive to movement artifacts than other brain imaging techniques. Over the last two decades, perception and cognition have been extensively studied in the cortical regions through fNIRS, which also allows studying functional connectivity among cortical regions (Boas et al., 2014; Homae, 2014). For example, Buss et al. (2014) showed that fNIRS can be used to study the frontal-parietal network at the base of visual working memory abilities. Similar to other neuroimaging techniques such as fMRI, a growing number of fNIRS studies use mini-block or event-related paradigms rather than block designs (Aqil et al., 2012; Aarabi et al., 2017). In fact, even if a block design significantly improves statistical power, mini-block or event-related paradigms crucially avoid strong habituation effects in the HRF time course of complex cognitive processes (Tie et al., 2009). In the present study, we aimed to advance knowledge on the use of fNIRS in complex cognitive paradigms relying on mini-block design by evaluating its use in emotional evaluation paradigms, which previous work suggested could constitute a relevant field to evaluate the suitability of fNIRS.

fNIRS has indeed recently proven a useful non-invasive technique to study emotion processes (Doi et al., 2013), especially in the visual domain (for a review, see Bendall et al., 2016). In one study, fNIRS was used to study affective processing of pictures in the parietal and occipital areas (Köchel et al., 2011); together with more recent work, it suggests that a large occipital-parietal-temporal network is involved in discrimination tasks involving judgments about ‘emotional’ gait patterns (Schneider et al., 2014). fNIRS has also allowed researchers to record prefrontal (PFC) activations during two types of task: the passive viewing and the active categorization of emotional visual stimuli. In the first case, researchers found an increase of the Oxy-Hb in the bilateral ventrolateral PFC when participants were watching negative pictures; in contrast, positive pictures led to a decrease of Oxy-Hb in the left dorsolateral PFC (Hoshi et al., 2011). In the second case, the authors isolated an activation of the bilateral PFC involving an increase of Oxy-Hb and a decrease of Deoxy-Hb when participants were viewing fearful rather than neutral images (Glotzbach et al., 2011). These results are consistent with recent findings showing fNIRS activations in ventrolateral PFC during the viewing of threatening pictures (Tupak et al., 2014). Finally, in a recent study, Hu et al. (2019) showed that fNIRS was suitable to isolate the signature of various positive emotions in the PFC. However, some studies did not find differences in Oxy-Hb between baseline and any kind of pictures, whether negative, neutral or positive (Herrmann et al., 2003). A natural negative mood during task completion was also found to have an impact on PFC activity during a working memory task (Aoki et al., 2011), although an experimentally induced negative mood had the opposite effect with increased PFC Oxy-Hb (Ozawa et al., 2014). As of now, the emerging picture for affective visual stimuli is that the PFC is solicited during both passive and active stimulation; however, the exact pattern of activity must be characterized with more studies and with an effort toward more comparability between the paradigms employed across fNIRS studies (Bendall et al., 2016).

While fNIRS studies are found in the literature with respect to visual emotional treatment, studies on affective processes using auditory signals remain rare in fNIRS research. That auditory emotional treatment is neglected is a concern given the abundance of work finding different cortical activations during auditory emotional evaluation through various imaging techniques. Indeed, even if much of the initial vocal emotional processing in the brain occurs in subcortical and sensory cortical areas (for a review, see Frühholz et al., 2014; Pannese et al., 2015), many higher order processes occur in cortical areas, including the associative temporal and the prefrontal cortices (Wildgruber et al., 2004; Frühholz et al., 2012; Frühholz and Grandjean, 2013a, b; Belyk et al., 2017). For example, in recent years, the PFC has been largely suggested to be involved in the processing of emotional stimuli in the vocal and auditory domain, based on work conducted mainly with fMRI (Frühholz and Grandjean, 2012; Dricu and Fruhholz, 2016; Frühholz et al., 2016a). In particular, the inferior frontal gyrus (IFG) is involved in the processing of human vocal sounds, and reacts to some of its properties such as prosody, the variation in intonations that modulates vocal production (Schirmer and Kotz, 2006; Frühholz et al., 2012). In a recent meta-analysis, Belyk et al. (2017) have reviewed the role of the *pars orbitalis* of the IFG during semantic and emotional processing, highlighting a possible functional organization in two different zones. The lateral one, close to Broca’s area, would be involved in both semantic and emotional aspects while the ventral frontal operculum would be more involved in emotional processing *per se*. The lateral zone would have been co-opted in human communication for semantic aspects while in non-human primates this zone would be more related to emotional communication. While we broadly agree with this view, the potential existence of vocalizations with semantic content in non-human primates (Gruber and Grandjean, 2017; Crockford et al., 2018) suggests that this co-optation may have emerged earlier in our evolution.

To our knowledge, only two studies have been published on the treatment of vocal emotional stimuli in fNIRS, both showing that emotional stimuli activated the auditory cortex more compared to neutral stimuli (Plichta et al., 2011; Zhang et al., 2018). While Plichta and colleagues did not investigate how vocal emotional stimuli modulated the activity in the PFC, Zhang and colleagues showed that the left IFG was modulated by emotional valence (positive vs. negative) and they also found a bilateral activation for the orbito-frontal cortex when anger was contrasted with neutral stimuli. However, neither of these two studies investigated categorization and discrimination of vocal emotional stimuli. To fill this gap, the present study investigated Oxy-Hb and Deoxy-Hb changes after the judgment of the emotional content of vocal utterances, with the aim to compare our results with recent fMRI advances. In particular, because of its involvement in the processing of human prosody, we aimed to target the IFG as our region of interest (ROI) in the present study.

An additional interesting aspect of the IFG is that this region is involved in both implicit and explicit categorization and discrimination of emotions in auditory stimuli. Implicit processing occurs when participants are required to conduct a task (e.g., judging the linguistic content of words or sentence pronounced with different emotional tones) other than evaluating the emotional content of the stimuli (e.g., Fecteau et al., 2005; Frühholz et al., 2012). The IFG is also involved when participants make explicit judgments (e.g., categorizing anger vs. fear) about the emotional content of the stimuli they are exposed to Ethofer et al. (2006), Mitchell (2006), Beaucousin et al. (2007), and Frühholz et al. (2016b). The right IFG may be particularly important for conducting such an explicit evaluation of the emotional content of the voices, although both hemispheres play a role in the processing of the emotional content (Frühholz and Grandjean, 2013). In general, independently of the implicit or explicit characteristic of the task, hemisphere biases for IFG activation can be expected in the evaluation of auditory emotional stimuli. For example, the right IFG appears especially activated during the listening of emotional stimuli (Wildgruber et al., 2004). In comparison, activations of the left IFG have been connected to the semantic content of a given vocal utterance, in part because the left IFG encompasses Broca’s area, which is particularly involved in speech processing (Friederici, 2012), and which the linguistic structure of pseudo-words (e.g., ‘belam’ or ‘molem’) used in auditory emotional paradigms is likely to trigger (Frühholz and Grandjean, 2013). Nevertheless, this lateralized view of the activity of the IFG is not shown in all studies. Indeed, several studies on emotional processing have found bilateral activations of the IFG (Kotz et al., 2003; Ethofer et al., 2009; Frühholz et al., 2012), or even left activations of specific areas of the IFG (Wildgruber et al., 2004; Fecteau et al., 2005; Bach et al., 2008) during emotional tasks. This suggests that different areas of the two IFGs are involved in different tasks concerned with the treatment of emotional vocal stimuli (Frühholz and Grandjean, 2013).

Despite the current caveats of the research on categorization and discrimination of auditory stimuli that we have outlined here, the well-established paradigms in fMRI as well as the extended literature make a strong case to transfer, adapt, and extend (by adding new emotional stimuli) the fMRI protocols to fNIRS. At the behavioral level, we expected to replicate results from the literature, that is participants would be more successful in discrimination compared to categorization, particularly in the pseudoword recognition compared to emotions (Dricu et al., 2017). At the brain level, in line with previous fNIRS studies in the visual modality, (i) we first predicted that active evaluation (categorization and discrimination) of auditory emotional stimuli would increase more Oxy-Hb changes in IFG compared to passive listening of the same stimuli. In addition, based on findings in fMRI (e.g., Dricu et al., 2017), we predicted that categorization (processing A-versus-B computations) would lead to more Oxy-Hb changes in IFG because it is cognitively more demanding than discrimination (only processing A-versus-Non-A computations). Second, based on the body of work in fMRI relying on implicit or explicit judgments, we predicted that (ii) Oxy-Hb changes would be modulated differentially according to the experimental manipulation of both the task (categorization or discrimination) and the content focus (condition: pseudoword or emotion). Finally, we also expected to capture hemisphere effects, based on the literature. Yet, because of the large variation recorded in the literature as reviewed above, we only hypothesized (iii) that emotional stimuli would involve more the right IFG than neutral stimuli but we did not produce strong hypotheses regarding hemisphere biases beforehand.

## Materials and Methods

### Participants

Twenty-eight healthy volunteers (14 males; mean age 26.44 years, *SD* = 4.7, age range 21–35) took part in the experiment. The participants reported normal hearing abilities and normal or corrected-to-normal vision. No participant presented a neurological or psychiatric history, or a hearing impairment. All participants gave informed and written consent for their participation in accordance with the ethical and data security guidelines of the University of Geneva. The study was approved by the Ethics Cantonal Commission for Research of the Canton of Geneva, Switzerland (CCER).

### Stimuli

The stimulus material consisted of three speech-like but semantically meaningless two-syllable pseudowords (i.e., “minad,” “lagod,” “namil”). These three stimuli were selected before the experiment from a pre-evaluation of a pool of pseudowords enounced on five emotion scales (sadness, joy, anger, fear, neutral) because they were most consistently evaluated as angry, fearful, and neutral, respectively (Frühholz et al., 2015 and see Supplementary Material). These pseudowords were 16-bit recordings sampled at a 44.1 kHz sampling rate. Two male and two female speakers spoke these three different pseudowords in an angry, fearful, or neutral tone, resulting in a total of 36 individual stimuli used in the current study. While there were individual differences between the speakers, all stimuli were evaluated by listeners (*N* = 12) as reflecting the correct emotion (Frühholz et al., 2015).

### Procedure

**FIGURE 1:**
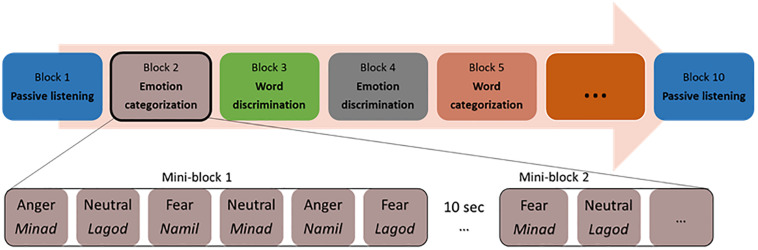
Experimental protocol with a possible list of blocks and stimuli within a mini-block.

Participants sitting in front of a computer performed two alternative forced-choice tasks of auditory discrimination and categorization via pressing a button on the keyboard. Stimuli were presented binaurally through in-ear headphones (Sennheiser). The participants listened to each voice and made a corresponding button press as soon as they could identify the requested target for each block. The categorization and discrimination blocks were split into blocks with a focus on emotion and blocks with a focus on the linguistic features of the stimuli. That is, either the participant had to select the pseudoword that they believed they heard, or the emotional tone with which it was pronounced. For discrimination, participants had to answer to a A vs. non-A question (e.g., “minad” vs. “other” or “fear” vs. “other”), while for categorization, participants had to answer a A vs. B question (“minad” vs. “lagod” vs. “namil or “fear” vs. “anger” vs. “neutral”). In the following and for simplicity, we will refer to all blocks concerned with the recognition of pseudoword as ‘word categorization’ or ‘word discrimination.’ Similarly, we will refer to all blocks concerned with the recognition of emotion as ‘emotion categorization’ or ‘emotion discrimination.’ Our experiment was thus blocked by tasks, based on a two (task: discrimination/categorization) by two (condition: emotion/word) design, with two blocks per condition and task (two each for emotion categorization, word categorization, emotion discrimination, and word discrimination). This allowed us to repeat each condition at least once and make sure that the data of at least one block could be analyzed if data acquisition came to a halt in a given block because of a software bug, which a pilot study suggested could occur. The eight blocks were preceded and followed by passive listening blocks, leading to 10 blocks in total (Figure 1). During the two passive blocks, participants only had to listen to the same stimuli as in the active tasks without having to make an active decision. Button assignments, target button and target stimuli alternated randomly across blocks for each participant. Task blocks, block order and response buttons also alternated through the experiment across participants, so that every participant had a unique ordering.

The two blocks of emotion categorizations involved a three-alternative forced-choice determining whether the speaker’s voice expressed an “angry,” “fearful,” or “neutral” tone (the options “angry” and “fear” were assigned to left and right index finger buttons, the “neutral” option included a simultaneous press of the left and right buttons no more than 500 ms apart).

The two blocks of word categorization involved a three-alternative forced-choice determining whether the pseudoword spoken was “minad,” “lagod,” or “namil” (the options “minad” and “lagod” were assigned to left and right index finger buttons, the “namil” option included a simultaneous press of the left and right buttons no more than 500 ms apart).

The discrimination blocks included a target emotion or a target pseudoword, which was assigned to one of the two response buttons. During the two emotion discrimination blocks, either angry or fearful voices were the target (e.g., press the left button for “angry” voices, and the right button for all other voices) and the two word discrimination blocks included either “minad” or “lagod” as the target pseudoword (e.g., press the left button for “minad,” and the right button for all other words). We acknowledge that by doing so, participants never had to discriminate “neutral” or “namil” against the opposite pseudowords or emotions. Testing all three would have required three blocks in each condition, multiplying the duration of the experiment or biasing it toward discrimination. In addition, by having “namil” and “neutral” always connected to the same behavioral response, we limited the possible number of button attribution errors (when a participant wrongly associates a button with a pseudoword or emotion, resulting in a stream of incorrect choices in a block), which would have likely increased if no single pseudoword or emotion had been bounded to a particular button combination.

Within each block, all 36 voice stimuli were presented twice resulting in 72 trials per block. These 72 trials were clustered into mini-blocks of six voice stimuli, where a stimulus was presented every 2s; each mini-block thus had an average length of 11.5–12 s. The presentation of mini-blocks was separated by 10s blank gap for the Oxy-Hb signal to return to baseline. Trials for each mini-block were randomly assigned, with the only exception that every emotion (with no more than three times the same emotion in a row) and every pseudoword had to appear at least one time per mini-block. Each mini-block started with a visual fixation cross (1 × 1°) presented on a gray background for 900 ± 100 ms. The fixation cross prompted the participant’s attention and remained on the screen for the duration of the mini-block.

### NIRS Recordings

**FIGURE 2:**
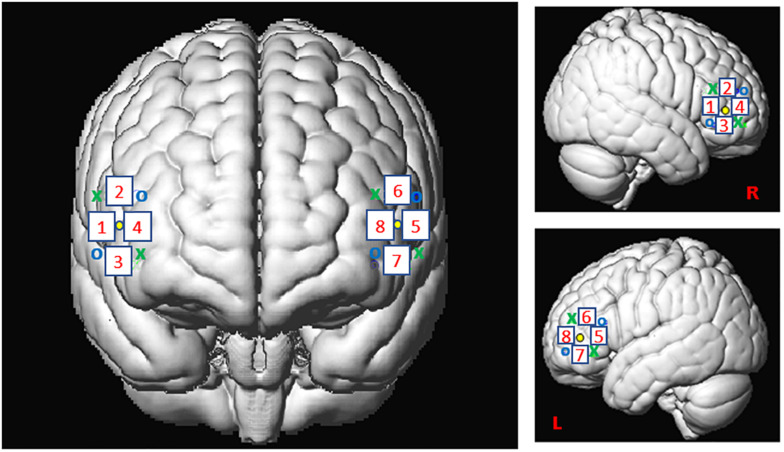
Spatial registration of optode locations to the Montreal Neurological Institute (MNI) space using spatial registration approach (Tsuzuki et al., 2007). This method relies on structural information from an anatomical database to estimate the fNIRS probe locations into a 3D space. Thus, this procedure allows the projection of the eight channels in the subject space into the MNI (Okamoto et al., 2004). Central dots indicate the F7 and F8 electrode position in the 10–20 EEG system. “o” and “x” indicate optical transmitter and receiver positions, respectively.

For this study, we used the Oxymon MKIII device (Artinis Medical Systems B.V., Elst, Netherlands) with a 2x4 optode template and wavelengths of 765 and 855 nm corresponding to an optimal range of signal to noise ratio (SNR, see Scholkmann et al., 2014). We placed four optodes as a square on both sides of the participant’s head, forming 4 channels around the F7 or F8 references and corresponding, respectively, to the left and right IFG (Figure 2), as defined in the 10-20- EEG system (Jasper, 1958; Okamoto et al., 2004). All channels were placed at an inter-optode distance of 35 mm and we recorded with a sampling rate of 250 Hz.

### Analysis

#### Behavioral Data

We only analyzed data from *N* = 26 participants (2 excluded for missing too many blocks) using R studio software [R Studio Team (2015) Inc., Boston, MA, United States^1^ ]. The accuracy analysis was performed on a total number of trials of *N* = 14’544 across the 26 participants (average: 559.39, *SD*: 42.27; on a basis of 576 trials/participant but with four participants’ dataset incomplete due to technical issues). We assessed accuracy in the tasks by predicting a generalized linear mixed model (GLMM) with binomial error distribution, with condition (emotion vs. word), task (categorization vs. discrimination), and emotion (anger, fear, neutral) as well as their interactions as fixed factors, and with intercept participant IDs and blocks (first or second) as random factors, against a GLMM with the same factors but not including the interaction between condition/task/emotion, allowing us to assess the effect of the triple interaction (see Supplementary Material for an example of model analysis). Note that for some models we used an optimizer to facilitate convergence. This analysis was followed by contrasts for which *post hoc* correction for multiple comparisons was applied by using a Bonferroni correction (0.05/66 = 0.00076). The specific contrasts we tested aimed to decipher whether the condition, emotion, and task had an effect on participants’ behavior. We analyzed reaction times by predicting a general linear mixed (GLM) model with condition (emotion vs. word), task (categorization vs. discrimination),and emotion (anger, fear, neutral), as well as their interactions as fixed factors, and with participant IDs and blocks as random factors using the same approach as in the analysis for accuracy. All reaction times were collected from the offset of the stimulus. We only included in our analyses the reaction times for correct answers. This resulted in a total number of trials of *N* = 13’789 across the 26 participants (average: 530.35, *SD*: 50.27). We excluded data points considered as outliers under 150 ms and higher than thrice the standard deviation (RT < 150 ms and >1860 ms; 98.85% of RT data points included).

#### fNIRS Data

Seven participants out of 28 were excluded from the dataset due to poor signal quality or missing fNIRS data. The absence or the low signal of heart beats in raw Oxy-Hb as well as a strong negative correlation between Oxy-Hb and Deoxy-Hb constituted a bad SNR. Furthermore, the presence of artifacts after band-pass filtering was also a factor of exclusion. A total of 21 participants were thus analyzed in this study. The number of participants was in line with statistical power analyses in fMRI (Desmond and Glover, 2002) and studies using fNIRS to assess emotional processing in frontal areas (for a review, see Bendall et al., 2016). Due to a good repartition of the SNR, we performed on all channels the first level analysis with MATLAB 2016B (Mathwortks, Natick, MA, United States) using the SPM_fNIRS toolbox (Tak et al., 2016^2^) and homemade scripts. Hemoglobin conversion and temporal preprocessing of Oxy-Hb and Deoxy-Hb were made using the following procedure:

(i) hemoglobin concentration changes were calculated with the modified Beer-Lambert law (Delpy et al., 1988);
(ii) motion artifacts were reduced using the method proposed by Scholkmann et al. (2010) based on moving standard deviation and spline interpolation;
(iii) physiological and high frequency noise such as due to vasomotion or heart beats usually found in extra-cerebral blood flow were removed using a band-stop filter between 0.12–0.35 and 0.7–1.5 Hz following Oostenveld et al. (2011) and a low-pass filter based on the HRF (Friston et al., 2000);
(iv) fNIRS data were down-sampled to 10 Hz;
(v) low frequency confound were reduced using a high-pass filter based on a discrete cosine transform set with a cut-off frequency of 1/64 Hz (Friston et al., 2000).

In line with previous literature using vocal stimuli in fNIRS studies (e.g., Lloyd-Fox et al., 2014), we considered the hemodynamic time course in our second level analyses. To select the range of the maximum concentration changes (μM) observed across participants for each trial, we averaged the concentration of Oxy-Hb between 4 and 12 s post-stimulus onset. As in fMRI studies, this interval took into consideration the slow timing of participants’ HRF and allowed us to assess precisely the Oxy-Hb concentration of one specific stimulus. We performed the same analyses on Deoxy-Hb to check our Oxy-Hb concentration changes (μM) for consistency. Because our results with Deoxy-Hb were coherent with the Oxy-Hb (Tachtsidis and Scholkmann, 2016), we only provide our results for Oxy-Hb in the main text (correlation coefficient: −0.97, *p* < 0.001, *N* = 12, Supplementary Figure S2; and see Supplementary Material for Deoxy-Hb analyses). All data were log-transformed to normalize them for the analyses.

We performed the second level analysis with R studio using Linear Mixed Models analysis including the following factors and their interactions depending on their pertinency in regard to our hypotheses (that is, we only run the contrasts that tested these hypotheses, rather than all the possible contrasts indiscriminately): condition (emotion vs. word), emotion content (anger vs. fear vs. neutral), task (categorization vs. discrimination vs. passive) and hemisphere (right vs. left, by pulling together data from channels 1–4 for the right hemisphere and data from channels 5–8 for the left hemisphere) as well as their interactions as fixed factors, with participant IDs and block orders as random factors. In particular, we predicted models including a higher-level interaction against models of the lower dimension (e.g., a four-way versus a three-way interaction + the main effects), presented in the results, on which we ran subsequent contrasts (see Supplementary Material for models with lower dimension interactions).

#### Analyses Including Passive Blocks

We first aimed to isolate whether our ROIs were activated differently during active blocks compared to passive blocks, in line with our first hypothesis (i). To do so, our first analyses confronted data collected during the passive and the active blocks. We were particularly interested in testing the effects of lateralization and emotional content, as previous fMRI studies had shown possible variation for these factors (see above). We noticed *post hoc* that subjects’ activations during the first and the final passive run differed widely, with the activation pattern found during the final passive run close to the pattern of activation recorded during the active tasks {see Supplementary Material, in particular Supplementary Figure S1, where we revealed a significant interaction of task by block number [χ^2^(2) = 2388.50, *p* < 0.001], with a significant contrast Passive 1 ^∗^ Passive 2: [χ^2^(1) = 4.33, *p* < 0.001]}. Therefore, it is likely that subjects were still engaged, consciously or not, in the discrimination or categorization of stimuli during the final passive block, even though they were instructed not to do so. For this reason, we excluded data from the final passive block, and only included data from the first passive block, for which no instruction besides listening to stimuli had been conveyed to the participants, ensuring their naivety to the task. To isolate any effect of active processes (that is processes occurring during blocks where the task was either discrimination or categorization) vs. passive processes, we tested a three-way model including data from the first passive run and all discrimination and categorization blocks. We specifically tested effects of active vs. passive blocks across emotions and hemispheres (iii), resulting in testing a three-way interaction between process (active vs. passive tasks), emotion (anger vs. fear vs. neutral) and hemispheres (right vs. left).

#### Analyses on Active Blocks Only

Second, in line with our second hypothesis (ii), we were interested in whether there were differences in activations between categorization or discrimination of words and emotions across hemispheres, and whether this depended on the emotion being tested. To do so, we focused on active blocks (discrimination and categorization blocks) and excluded the passive blocks, as the subjects had no specific instructions regarding the stimuli compared to the active blocks (see above). To isolate any differences between the factors, we tested a four-way interaction on the active blocks including the effects of hemisphere (right vs. left), tasks (discrimination vs. categorization), conditions (word vs. emotion), and emotions (anger vs. fear vs. neutral). Subsequently, as in our first analysis, we tested contrasts between right and left hemispheres. In a final analysis, we individually looked at each hemisphere (iii) to contrast anger, respectively, fear, versus neutral stimuli.

## Results

### Behavioral Data

#### Accuracy Data

**FIGURE 3:**
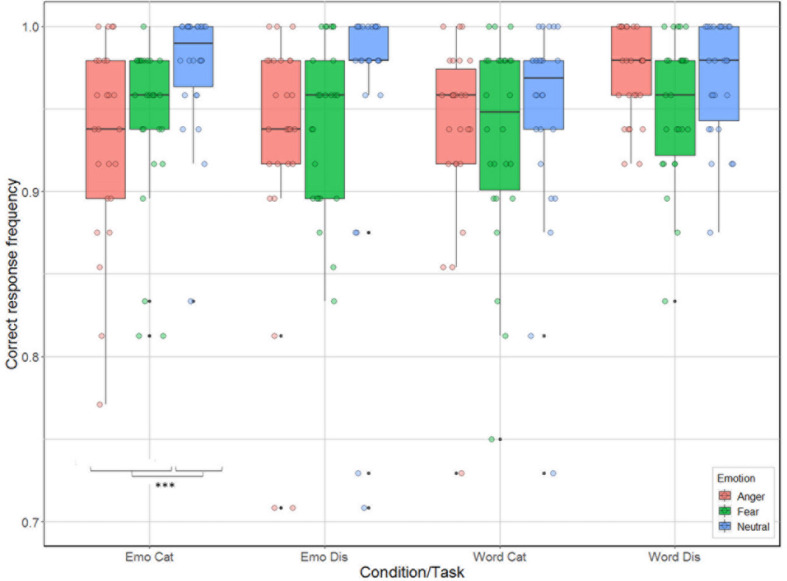
Accuracy (in %) of the 26 participants represented as a function of condition (emotion vs. word) and task (categorization vs. discrimination), and emotion (anger, fear, neutral).

There were significant effects for task [categorization vs. discrimination; χ^2^(1) = 6.38, *p* = 0.012], and emotion [anger, fear, neutral; χ^2^(2) = 33.01, *p* < 0.001], and for the interactions condition by task [χ^2^(1) = 21.17, *p* < 0.001], and condition by emotion [χ^2^(2) = 14.00, *p* < 0.001], but not for the main effect related to condition [emotion vs. word; χ^2^(1) = 2.54, *p* = 0.11] and for the interactions task by emotion [χ^2^(2) = 4.65, *p* = 0.098], and task by condition by emotion [χ^2^(2) = 2.31, *p* = 0.32]. Analysis of the contrasts of interest, following Bonferroni correction, revealed that participants were better for categorization when listening to neutral compared to anger and fear stimuli for the emotion condition [neutral vs. anger: χ^2^(1) = 28.42, *p* = 0.0004; neutral vs. fear: χ^2^(1) = 15.06, *p* = 0.0001; Figure 3]. This effect was not present for emotion discrimination [neutral vs. anger: χ^2^(1) = 9.42, *p* = 0.002; neutral vs. fear: χ^2^(1) = 8.47, *p* < 0.004], nor for categorization (*p*-values > 0.2) or discrimination [neutral vs. anger: χ^2^(1) < 0.01; neutral vs. fear: χ^2^(1) = 5.44, *p* = 0.02] in the word condition.

#### Reaction Time

**FIGURE 4:**
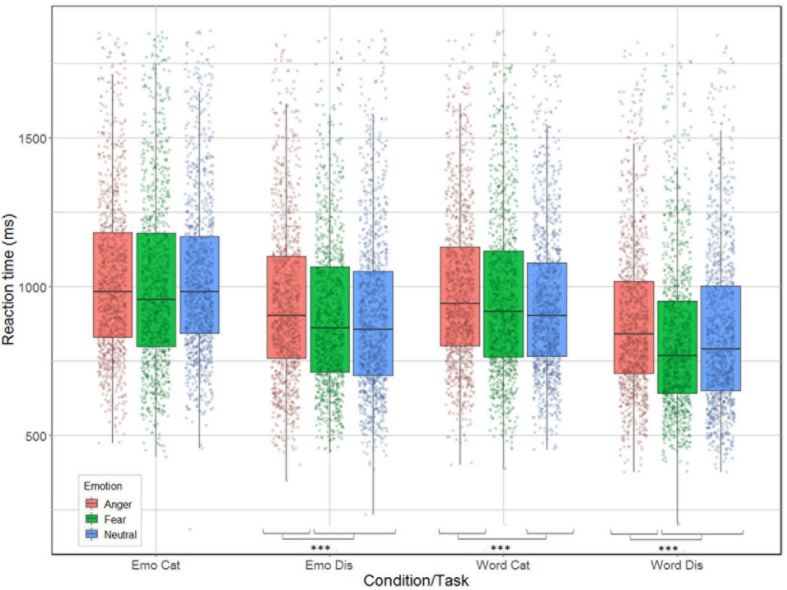
Reaction time (in ms) for the correct trials of the 26 participants represented as a function of condition (emotion vs. word) and task (categorization vs. discrimination), and emotion (anger, fear, neutral).

Correlation between reaction time and accuracy was extremely weak (Spearman’s rho = 0.033). The analysis revealed significant main effects for condition [χ^2^(1) = 240.51, *p* < 0.001], task [χ^2^(1) = 653.12, *p* < 0.001], and emotion [χ^2^(2) = 61.66, *p* < 0.001]; as well as significant interactions between task by emotion [χ^2^(2) = 9.83, *p* = 0.007] and between all three factors [χ^2^(2) = 14.2, *p* < 0.001] but not for condition by task [χ^2^(1) = 1.92, *p* = 0.17] nor condition by emotion [χ^2^(2) = 5.42, *p* = 0.07, see Figure 4]. Contrast analysis using the same Bonferroni correction as in the accuracy analysis revealed that participants were slower for anger compared to fear and neutral during emotion [respectively, χ^2^(1) = 15.46, *p* < 0.0001 and χ^2^(1) = 17.55, *p* < 0.0001], and word discrimination [respectively, χ^2^(1) = 41.65, *p* < 0.0001 and χ^2^(1) = 15.89, *p* < 0.0001]. For word categorization the comparison between anger/neutral was significant [χ^2^(1) = 21.15, *p* < 0.0001] but not for anger/fear [χ^2^(1) = 4.82, *p* = 0.028]. For emotion categorization both the comparisons anger/fear and anger/neutral were not significant (*p*-values > 0.026).

### NIRS Data

#### Analyses Including the First Passive Run

**FIGURE 5:**
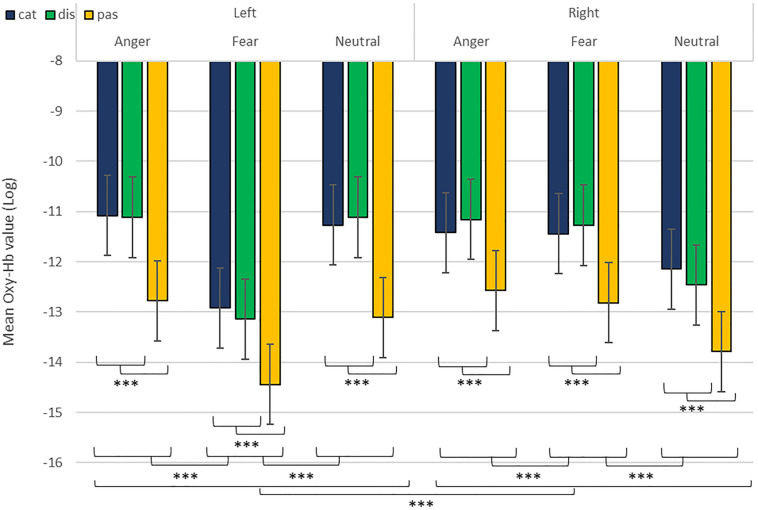
Contrast in log of Oxy-Hb concentration changes (μM) in the right and left hemispheres during the treatment of anger, fear, and neutral stimuli. ***p < 0.001.

**FIGURE 6:**
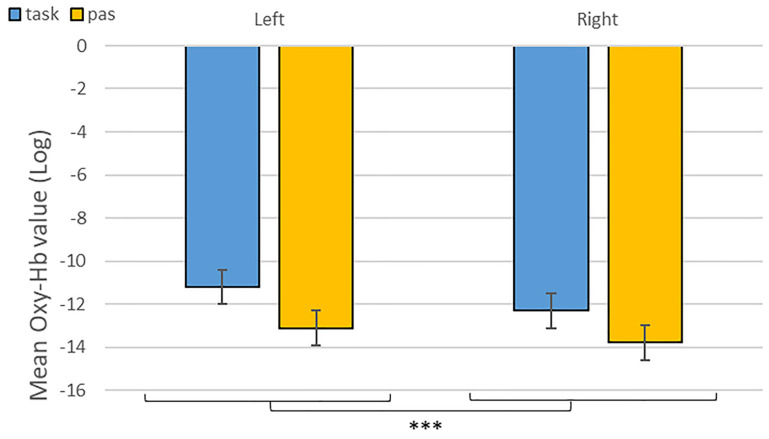
Contrast between log values of Oxy-Hb concentration changes (μM) for activities during passive listening and active (categorisation and discrimination) blocks for neutral stimuli only. ***p < 0.001.

As predicted, we revealed a significant three-way interaction of task by hemisphere by emotion [χ^2^(10) = 262.47, *p* < 0.001, see Table 1]. We subsequently ran contrasts to isolate the contributions of each of the factors. In particular, when contrasting passive listening vs. active tasks (categorization and discrimination) with lateralization (right vs. left) and pairs of emotions together, we found a significant difference with higher Oxy-Hb values for tasks vs. passive listening for ‘fear’ compared to ‘neutral’ on the right compared to left hemisphere [χ^2^(1) = 18.13, *p* < 0.001; Figure 5]; and a significant difference for ‘fear’ compared to ‘anger’ with higher Oxy-Hb for anger on the left compared to the right hemisphere [χ^2^(1) = 15.16, *p* < 0.001]; in comparison, ‘anger’ vs. ‘neutral’ did not yield significant differences [χ^2^(1) = 0.13, *p* = 0.72]. When only considering neutral stimuli, the contrast between passive listening and tasks was also significant with higher values for left compared to right [χ^2^(1) = 29.02, *p* < 0.001; see Figure 6], showing a general task difference independent of emotional content.

**TABLE 1:** Summary of the main effects and results of the three-way interaction between the factors in the models assessing passive vs. active processes (categorization, discrimination) comparison.

	χ^2^ value	Df	*p*
**Main effects**			
Hemisphere	93.4	1	<0.001
Emotion	2758.8	2	<0.001
Task	3491.6	2	<0.001
**Interaction**			
Task * Hemisphere * Emotion	262.47	10	<0.001
**Contrasts**			
Active vs. passive, anger vs. neutral, left vs. right	0.13	1	0.72
Active vs. passive, fear vs. neutral, left vs. right	18.13	1	<0.001
Active vs. passive, fear vs. anger, left vs. right	15.16	1	<0.001
Active vs. passive, only neutral, left vs. right	29.02	1	<0.001

#### Analyses of the Active Blocks

**FIGURE 7:**
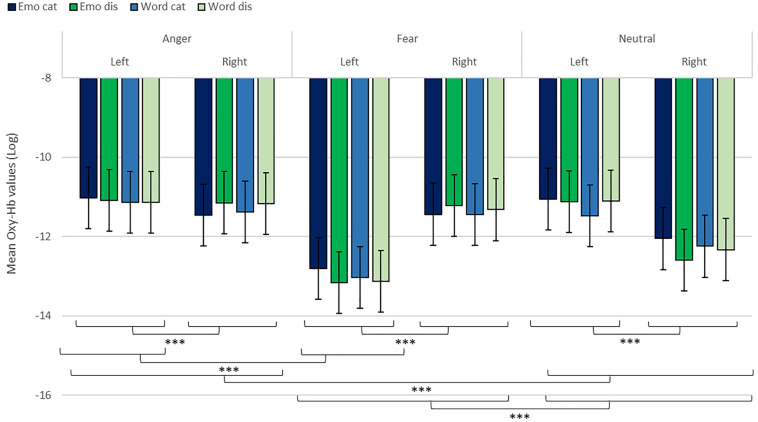
Contrast in log of Oxy-Hb concentration changes (μM) for anger, fear, and neutral stimuli in the right and left hemispheres for emotional categorization/discrimination and word categorization/discrimination. ***p < 0.001.

We revealed a significant four-way interaction of task by hemisphere by condition by emotion [χ^2^(11) = 117.04, *p* < 0.001, see Table 2], confirmed also for Deoxy-Hb [χ^2^(17) = 2463.9, *p* < 0.001, see Supplementary Material]. To test the specific significant effects related to emotions and lateralization, we performed the following contrasts: we tested the impact of condition (emotion versus word), hemisphere (left vs. right) and task (discrimination vs. categorization) for each emotion individually: anger [χ^2^(1) = 32.54, *p* < 0.001], fear [χ^2^(1) = 54.85, *p* < 0.001], and neutral [χ^2^(1) = 79.84, *p* < 0.001]. In addition, we also contrasted emotions with each other: the contrasts condition, hemisphere, task for anger vs. fear [χ^2^(1) = 1.45, *p* = 0.23] did not reach significance but the contrasts anger vs. neutral [χ^2^(1) = 107.16, *p* < 0.001] and fear vs. neutral did [χ^2^(1) = 133.52, *p* < 0.001, Figure 7], suggesting that the comparison across hemispheres between fear and neutral, on the one hand, and fear and anger, on the other hand, drove most of the interaction.

**TABLE 2:** Summary of the main effects and results of the four-way interaction between the factors in the models assessing the active tasks comparison.

	χ^2^ value	Df	*p*
**Main effects**			
Condition	14.27	1	<0.001
Hemisphere	58.98	1	<0.001
Emotion	2681.8	2	<0.001
Task	0.01	1	0.92
**Interaction**			
Task * Hemisphere * Condition * Emotion	117.04	11	<0.001
**Contrasts**			
Task * hemisphere * condition (anger only)	32.54	1	<0.001
Task * hemisphere * condition (fear only)	54.85	1	<0.001
Task * hemisphere * condition (neutral only)	79.84	1	<0.001
Task * hemisphere * condition (anger vs. fear)	1.45	1	0.23
Task * hemisphere * condition (anger vs. neutral)	107.16	1	<0.001
Task * hemisphere * condition (fear vs. neutral)	133.52	1	<0.001
**Contrasts per hemisphere**			
Task * hemisphere (right) * condition (anger vs. neutral)	120.48	1	<0.001
Task * hemisphere (left) * condition (anger vs. neutral)	13.42	1	<0.001
Task * hemisphere (right) * condition (fear vs. neutral)	83.83	1	<0.001
Task * hemisphere (left) * condition (fear vs. neutral)	51.63	1	<0.001
Task * hemisphere (right) * condition (anger vs. fear)	3.31	1	0.069
Task * hemisphere (left) * condition (anger vs. fear)	12.40	1	<0.001

Finally, to investigate the specificities of the lateralization, we also ran contrasts on the left or right hemispheres only (Table 2). This analysis revealed a significant effect of ‘anger’ vs. ‘neutral’ on the left hemisphere [χ^2^(1) = 13.42, *p* < 0.001]; this effect was also significant for the right hemisphere [χ^2^(1) = 120.48, *p* < 0.001]. The comparison for ‘fear’ vs. ‘neutral’ was also significant for both the left [χ^2^(1) = 51.63, *p* < 0.001] and right hemispheres [χ^2^(1) = 83.83, *p* < 0.001]. Finally, the comparison for ‘fear’ vs. ‘anger’ was significant for the left [χ^2^(1) = 12.4, *p* < 0.001, Figure 7] but not the right hemisphere [χ^2^(1) = 3.31, *p* = 0.07].

## Discussion

In this study we showed that fNIRS is a suitable method to study cognitive paradigms related to emotions, particularly categorization and discrimination, in the human frontal regions using mini-block design and event related stimuli. Our first goal was to estimate whether it was possible to isolate significant activity in the IFG using fNIRS, whose activity has been highlighted in previous fMRI studies investigating emotional prosody processing, and in particular during categorization and discrimination of emotional stimuli (Schirmer and Kotz, 2006; Frühholz et al., 2012). Both the right and left IFGs have been connected to the processing of emotional stimuli (Wildgruber et al., 2004; Ethofer et al., 2006; Frühholz et al., 2012) and we were interested to investigate such effects in more depth with fNIRS. We predicted (i) that active evaluation (categorization and discrimination) of auditory emotional stimuli would increase more Oxy-Hb changes in IFG compared to passive listening of the same stimuli, and that categorization itself would be more demanding than discrimination, which would be both reflected in the brain and behavioral data. Our second goal was to investigate whether fNIRS, beyond being suitable, could also offer informative data in complex multifactorial analyses. In particular, we expected (ii) that the Oxy-Hb changes would be modulated differentially according to the tasks, conditions and emotions, with the possible presence of hemisphere biases.

Overall, we found increased differential changes in Oxy-Hb in the IFG based on experimental conditions suggestive of significant differences in frontal activations during our tasks, including a difference in activation during categorization and discrimination compared to passive listening in the Oxy-Hb and confirmed in the Deoxy-Hb signals. In particular, in our first analysis of the NIRS signal, we isolated left hemisphere activity for active processing versus passive listening of neutral stimuli (Figure 6). This result suggests that fNIRS is in general a suitable method to identify brain signatures related to complex processes such as categorization and discrimination in auditory stimuli.

In addition, while we did not observe a main effect of task in the active-only analyses, we uncovered significant interactions that included task, condition and emotion content, suggesting that categorization and discrimination of various content have different fNIRS signatures, and underlining that fNIRS can be used in complex multifactorial paradigms. Furthermore, we isolated specific hemispheric differences between emotions that can be linked with findings in fMRI. While our study was primarily aimed at showing that fNIRS was suitable to use for the study of auditory discrimination and categorization, our results are also of interest in the current debate on the lateralization of effects in the brain, in particular when compared to former fMRI studies concerned with the involvement of the PFC in the evaluation of emotional stimuli (Ethofer et al., 2006; Dricu et al., 2017). When considering active and passive tasks, the effect for fear and anger versus neutral was more pronounced in the right hemisphere (Figure 5), in line with classic studies highlighting a right dominance for emotional treatment in prosody (Wildgruber et al., 2004) and our preliminary hypothesis (iii). However, while the left hemisphere was more deactivated with fear stimuli, anger stimuli activated more the left side of the prefrontal lobe compared to the right side. Both findings are compatible with Davidson’s view (Davidson, 2004), for whom approach-related emotions such as anger activate more the left hemisphere, particularly in the prefrontal cortex, while avoidance-related emotions, such as fear, are more located in the right hemisphere. Furthermore, our second analysis on active tasks only also revealed significant differences between categorization and discrimination between the experimental conditions: indeed, we found a significant four-way interaction between condition (word vs. emotion), task (categorization vs. discrimination), emotion and hemisphere. Interestingly, the results of the analysis of the contrasts suggest that differences in brain activity between categorization and discrimination and lateralization are more important for fear and anger stimuli compared to neutral ones, both on the right and left hemisphere. Nevertheless, activity for anger stimuli across conditions and tasks was higher compared to other stimuli (Figure 7). This result supports a bilateral approach to the treatment of emotional stimuli (Schirmer and Kotz, 2006; Frühholz and Grandjean, 2013b).

Our behavioral results are also informative with respect to a differential treatment of stimuli depending on emotion content, condition and task. While our participants were generally accurate across tasks and conditions (over 96% correct in all tasks), and while we cannot exclude that the minor but significant variations between the four experimental conditions result from the very large number of data points, which made the standard errors quite small, we note that these differences nevertheless appear to reflect the variations in treatment outlined in the four-way interaction found in the fNIRS data. Participants were most accurate when engaged in emotional categorization, seconded by word discrimination, with the lowest accuracy rates found for word categorization and emotional discrimination. This result may seem counter-intuitive at first, as categorization appears to be cognitively more difficult than discrimination. However, there was also much variation in terms of emotion recognition, with participants more accurate with neutral stimuli when their task was to categorize the correct emotional content. However, the difference across emotions was not present when their task was to judge the linguistic content of the words, nor when they had to discriminate emotions, possibly because of our experimental design. In addition, participants’ reaction times also varied between the conditions and emotions: overall, categorization took more time compared to discrimination, with judgments made on emotional content always taking longer than on linguistic content, particularly with respect to anger stimuli. This behavioral finding may reflect the increased activation across hemispheres observed in the fNIRS data for anger stimuli. Combined, these results suggest different processing between words and emotions (in line with Belyk et al., 2017), with active judgments on emotional stimuli being more demanding (longest reaction time) than judgments on the linguistic content. Indeed, when participants judged the emotional content of stimuli, they were more accurate for categorization than discrimination but spent a longer time before selecting their answer. In contrast, for words, participants were more accurate for discrimination compared to categorization, but they spent less time before answering.

Another potential explanation for the differences observed between the active processing of emotional aspects compared to linguistic aspects lies in the fact that the IFG is activated during both implicit and explicit categorization and discrimination of emotions (Fecteau et al., 2005; Ethofer et al., 2006; Mitchell, 2006; Beaucousin et al., 2007; Frühholz et al., 2012; Dricu and Fruhholz, 2016). Our participants may thus have engaged in implicit emotional processing of the stimuli even when their task was to judge the linguistic aspect of the stimuli. This additional treatment may explain the Oxy-Hb differences found between emotions even in the context of word categorization and discrimination. The right IFG has previously been highlighted as particularly important in the explicit evaluation of the emotional content of the voices, and our Oxy-Hb results support this view, particularly when considering fear versus neutral stimuli. The generally higher activity in both hemispheres when participants processed stimuli with an angry content also supports the view that both hemispheres play a role in the processing of the emotional content, whether implicit or explicit (Frühholz and Grandjean, 2013). Future work will need to explore the specific aspects of emotional stimuli when more types of emotion (e.g., positive) are included. It may also be interesting to study whether bilateral or unilateral treatments are elicited depending on the evaluation process, implicit or explicit.

In general, more work is needed to assess the limitations of fNIRS with respect to complex cognitive processing. For example, there is only an indirect link between the Oxy-Hb measures and the actual neural activity, which will eventually limit the direct connections that can be extrapolated between variation in activity in a given ROI and the behavior of participants. Note, however, that this criticism also applies to other techniques (e.g., fMRI) relying on indirect measures such as blood oxygen-level dependent signal to reflect neural activity (Ekstrom, 2010). In our view, work relying on different imaging techniques can thus only improve our understanding of this indirect relationship, and a possible new avenue of research is to combine fMRI and fNIRS to explore auditory evaluation of stimuli. It seems also mandatory at this stage to decipher what results from emotional processing from other auditory processing. For example, effortful listening has been shown to also affect activity in the PFC and IFG (Rovetti et al., 2019), something that our study did not account for. In particular, listening to emotional stimuli and pseudowords may be more effortful than listening to traditional speech and thus might have also driven some of the recorded effect. Future work using this type of paradigms will thus need to tackle other cortical activities related to processing auditory stimuli in general.

To conclude, our study shows that, despite its caveats, fNIRS is a suitable method to study emotional auditory processing in human adults with no history of psychiatric antecedents or hearing impairment. Beyond fNIRS studies investigating emotions from a perceptual point of view (e.g., Plichta et al., 2011; Zhang et al., 2018), our study replicates and extends effects found with more traditional imaging methods such as fMRI and shows that subtle differences can be found in fNIRS signal across tasks and modalities in the study of emotional categorization and discrimination. Future work will need to examine in more details whether differences between stimuli valence or arousal may also influence the fNIRS signal. In this respect, one of the major advantages of fNIRS lies in the fact that it is noiseless. This is all the more important for studies that investigate the perception of sounds, but also in general for more realistic experiments. fNIRS may also be very informative in the context of prosody production thanks to its resistance to movement artifacts compared to other brain imaging methods. Combined with its portability and ease of use, fNIRS may also extend such questions in populations where the use of fMRI is limited such as young infants, populations in less developed countries or, possibly, other species (Gruber and Grandjean, 2017). The use of unfamiliar non-verbal human or non-human vocalizations rather than pseudowords may be particularly informative to study the developmental and evolutionary origins of the two cognitive processes. Finally, our study contributes to the growing field of affective neurosciences, confirming through a different imaging technique that emotion treatment, both explicitly and implicitly, may be largely conducted in the IFG, a possible hub for the extraction and detection of variant/invariant aspects of stimuli (e.g., acoustical features) subjected to categorization/discrimination representation (e.g., anger/neutral prosody) in the brain.

## Data Availability Statement

The raw data supporting the conclusions of this article will be made available by the authors, without undue reservation, to any qualified researcher.

## Ethics Statement

The studies involving human participants were reviewed and approved by the Ethics Cantonal Commission for Research of the Canton of Geneva, Switzerland (CCER).

## Author Contributions

TG, DG, and SF designed the study. CD, TG, KI, and BM conducted the data acquisition. CD, DG, LC, TG, and SF analyzed the data. TG, DG, CD, SF, KI, and BM wrote the manuscript.

## Conflict of Interest

The authors declare that the research was conducted in the absence of any commercial or financial relationships that could be construed as a potential conflict of interest.

## Footnotes

1 http://www.rstudio.com/

2 https://www.nitrc.org/projects/spm_fnirs/
