# Supplementary material for: Human Discrimination and Categorization of Emotions in Voices: A Functional Near-Infrared Spectroscopy (fNIRS) Study
Source: Front Neurosci. 2020 Jun 5;14:570. doi: 10.3389/fnins.2020.00570 (PMC7290129; doi:10.3389/fnins.2020.00570)
Supplement: Supplementary file 1 [file Table_1.DOCX]

Supplementary Material

**Selection of stimuli for the experiment**

From a pre-evaluation of all stimuli on five emotion scales (sadness, joy, anger, fear, neutral), we selected three pseudowords for each emotional tone that were most consistently evaluated as angry (*F*_4,80_=256.111, *p*<0.001), fearful (*F*_4,80_=151.894, *p*<0.001), and neutral (*F*_4,80_=193.527, *p*<0.001), respectively ^46^. One-way ANOVA revealed that there was a statistically significant difference in arousal scores among the angry, fear, and neutral voices (*F*_2,40_=54.073, *p*<0.001). Bonferroni corrected post-hoc planned comparisons revealed that arousal scores for angry and fearful tones were significantly higher than scores received by neutral tones (*p*<0.001), but they did not differ significantly from each other (*p*=0.408).

**Example of R script analysis for accuracy**

Comparison of the model with the triple interaction and the model with main effects and double interactions including random effects (intercept) with binomial family:

model.lme1<glmer(Acc~Condition+Task+Emotion+Condition:Task+Condition:Emotion+Task:Emotion+Condition:Task:Emotion+(1|ID)+(1|Block), family=binomial (link = "logit"), glmerControl (optimizer = "bobyqa", optCtrl = list(maxfun = 1000000)), data=stim)

model.lme2<-glmer(Acc~Condition+Task+Emotion+Task:Condition+Condition:Emotion+Task:Emotion+ (1|ID) + (1|Block), family=binomial(link = "logit"), glmerControl(optimizer="bobyqa", optCtrl = list(maxfun = 1000000)), data=stim)

anova(model.lme1, model.lme2)

All other behavioral and NIRS analysis are based on similar scripts with generalized or general linear mixed models.

**Analyses first (active and passive) blocks versus second (active and passive) blocks**

Task * Block number

We revealed a significant interaction of task * block number (χ^2^(2)=2388.50, p<0.001).

Contrasts

Passive * active: (χ^2^(1)=2400.60, p<0.001)

Passive 1 * passive 2: (χ^2^(1)=4.334.10, p<0.001)


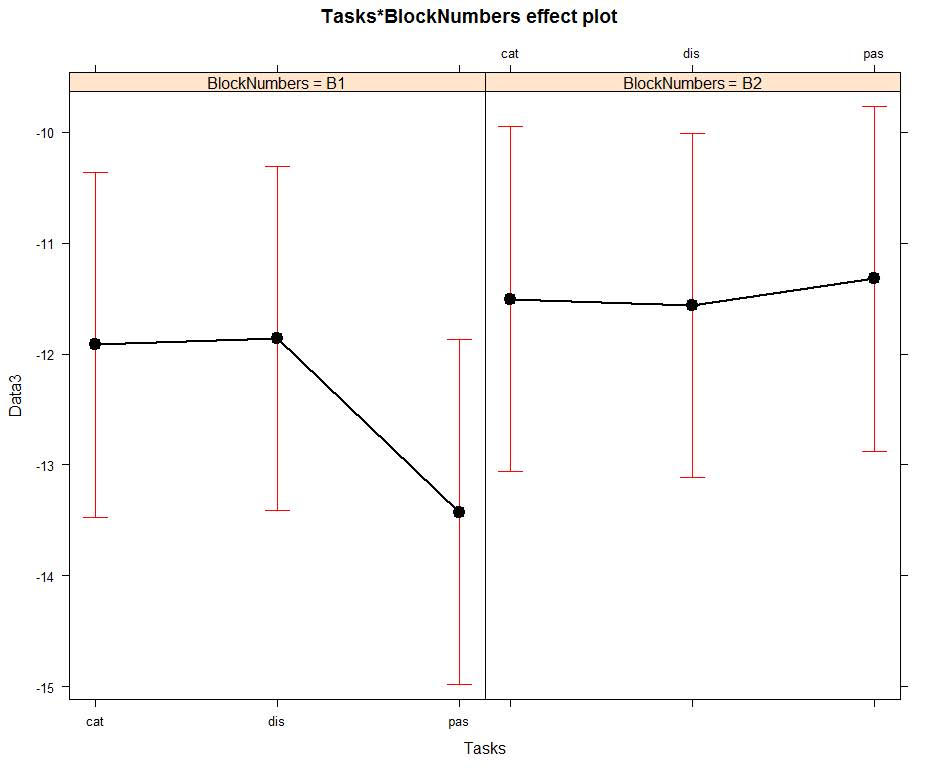


**Figure S1**. Contrast between log values of Oxy-Hb concentration changes (µM) for activities during the first (B1) and the second (B2) passive listening and active blocks including all stimuli.

**Analyses Oxy-Hemoglobin Signal**

*Analyses including the first passive run*

**Main effects**

Hemisphere

We revealed a significant main effect of hemisphere (χ^2^(1)=93.4, p<0.001).

Emotion

We revealed a significant main effect of emotion (χ^2^(2)=2758.8, p<0.001).

Task

We revealed a significant main effect of task (χ^2^(2)=3491.6, p<0.001).

**Interactions**

Task * Emotion

We revealed a significant two-way interaction of task * emotion (χ^2^(4)=48.8, p<0.001).

Task * Hemisphere

We revealed a significant two-way interaction of task * hemisphere (χ^2^(2)=33.4, p<0.001).

Emotion * Hemisphere

We revealed a significant two-way interaction of emotion * hemisphere (χ^2^(2)=5564.9 , p<0.001).

Task * Hemisphere* Emotion

We revealed a significant three-way interaction of task * hemisphere * emotion (χ^2^(10)=262.47, p<0.001).

*Analyses on active blocks*

**Main effects**

Condition

We revealed a significant main effect of condition (χ^2^(1)=14.27, p<0.001).

Emotion

We revealed a significant main effect of emotion (χ^2^(2)=2681.8, p<0.001).

Hemisphere

We revealed a significant main effect of hemisphere (χ^2^(1)=58.98, p<0.001).

Task

There was no significant effect of task (χ^2^(1)=0.01, p=0.92).

**Interactions**

Task * Hemisphere

We revealed a significant two-way interaction of task * hemisphere (χ^2^(1)=5.29, p<0.05).

Task * Condition

We revealed a significant two-way interaction of task * condition (χ2(1)=28.56, p<.001).

Emotion * Hemisphere

We revealed a significant two-way interaction of task * emotion (χ^2^(2)=5639, p<0.001).

Condition * Hemisphere

We revealed a significant two-way interaction of condition * hemisphere (χ^2^(1)=16.63, p<0.001).

Task * Hemisphere * Condition

We revealed a significant three-way interaction of task * hemisphere * condition (χ^2^(3)=40.9, p<0.001).

Task * Hemisphere * Emotion

We revealed a significant three-way interaction of task * hemisphere * emotion (χ^2^(5)=195.42, p<0.001).

**Analyses Deoxy-Hemoglobin Signal**


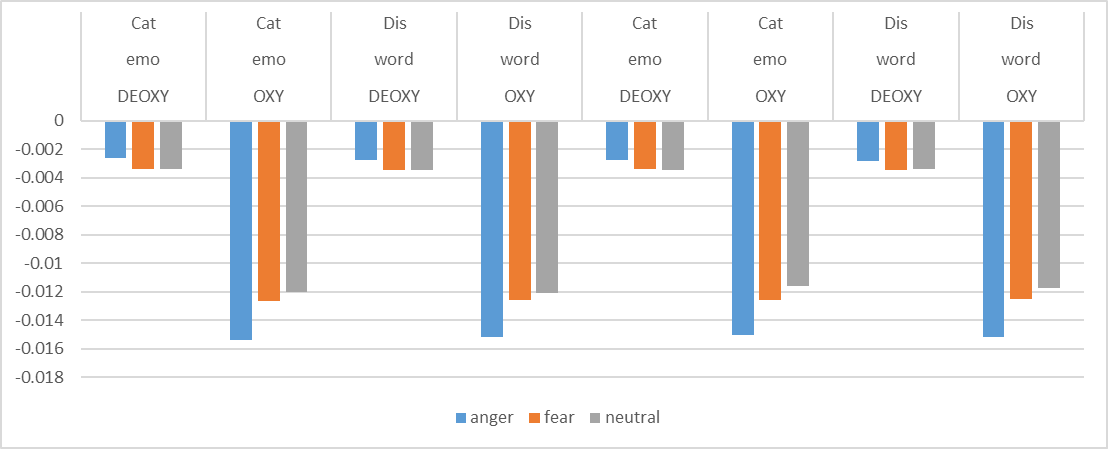


**Figure S2**. Average of Oxy-Hb and Deoxy-Hb concentration changes (µM) during categorization and discrimination tasks for each emotion. Correlation coefficient: -0.97, p<0.001.

*Analyses including the first passive run*

**Main effects**

Hemisphere

We revealed a significant main effect of hemisphere (χ^2^(1)=64.88, p<0.001).

Emotion

We revealed a significant main effect of emotion (χ^2^(2)=2575.7, p<0.001).

Task

We revealed a significant main effect of task (χ^2^(2)=1755, p<0.001).

**Interactions**

Task * Emotion

We revealed a significant two-way interaction of task * emotion (χ^2^(4)=152.92, p<0.001).

Task * Hemisphere

We revealed a significant two-way interaction of task * hemisphere (χ^2^(2)=32.41, p<0.001).

Emotion * Hemisphere

We revealed a significant two-way interaction of emotion * hemisphere (χ^2^(2)=2242.1, p<0.001).

Task * Emotion * Hemisphere

We revealed a significant three-way interaction of task * emotion * hemisphere (χ^2^(10)=316.92, p<0.001).

*Analyses on active blocks*

**Main effects**

Condition

We revealed a significant main effect of condition (χ^2^(1)=23.26, p<0.001).

Emotion

We revealed a significant main effect of emotion (χ^2^(2)=2612.9, p<0.001).

Hemisphere

We revealed a significant main effect of hemisphere (χ^2^(1)=43.53, p<0.001).

Task

We revealed a significant main effect of task (χ^2^(1)=80.67, p<0.001).

**Interactions**

Task * Hemisphere

We revealed a significant two-way interaction of task * hemisphere (χ^2^(7)=21.41, p<0.001).

Task * Condition

There was no significant effect of task * condition (χ^2^(1)=1.56, p=0.21).

Task * Emotion

We revealed a significant two-way interaction of task * emotion (χ^2^(2)=76.18, p<0.001).

Hemisphere * Condition

We revealed a significant two-way interaction of hemisphere * condition (χ^2^(1)= 8.12, p<0.005).

Task * Hemisphere * Emotion

We revealed a significant three-way interaction of task * hemisphere * emotion (χ^2^(6)=2388, p<0.001).

Task * Hemisphere * Condition

We revealed a significant three-way interaction of task * hemisphere * condition (χ^2^(3)= 30.02, p<0.001).

Task * Hemisphere * Condition * Emotion

We revealed a significant four-way interaction of task * hemisphere * condition * emotion (χ^2^(17)=2463.9, p<0.001).
